# Platelet-to-Albumin Ratio and Clinical Outcomes in IDH-Wildtype Grade 4 Diffuse Glioma

**DOI:** 10.3390/jcm15145512

**Published:** 2026-07-14

**Authors:** Ozan Deniz Guven, Asim Armagan Aydin, Ahmet Unlu, Hayrani Kaya, Fatma Su Ovali, Abdullah Umit, Murat Kocer, Banu Ozturk, Mustafa Yildiz

**Affiliations:** Department of Clinical Oncology, Antalya Training and Research Hospital, University of Health Sciences, 07100 Antalya, Turkey; md.ahmetunlu@gmail.com (A.U.); hayrani_kaya@outlook.com (H.K.); fsudemir@gmail.com (F.S.O.); docabdullah036@gmail.com (A.U.); muratkocer71@hotmail.com (M.K.); drbanutr@yahoo.com (B.O.); drmyildiz@yahoo.com (M.Y.)

**Keywords:** platelet-to-albumin ratio, neuro-oncology, glioblastoma, tumor–host interaction, systemic inflammation, IDH-wildtype grade 4 diffuse glioma, MGMT promoter methylation, progression-free survival, prognostic biomarker, overall survival

## Abstract

**Background:** Clinical outcomes in isocitrate dehydrogenase (IDH)-wildtype grade 4 diffuse glioma remain highly heterogeneous despite standard multimodal therapy. We evaluated the prognostic significance of pretreatment platelet-to-albumin ratio (PAR) and compared its performance with established inflammatory biomarkers. **Methods:** This retrospective cohort study included 166 patients with histopathologically confirmed IDH-wildtype grade 4 diffuse glioma treated between 2017 and 2025. Pretreatment PAR, neutrophil-to-lymphocyte ratio (NLR), systemic immune-inflammation index (SII), systemic inflammation response index (SIRI), pan-immune inflammation value (PIV), C-reactive protein-to-albumin ratio (CAR), and lactate dehydrogenase-to-albumin ratio (LAR) were evaluated. Discriminative performance was assessed using classical and inverse probability of censoring weighting (IPCW)-adjusted time-dependent receiver operating characteristic (ROC) analyses. Survival outcomes were evaluated using Kaplan–Meier analyses and predefined baseline-adjusted multivariable Cox proportional hazards regression models. Internal validation was performed using 1000 bootstrap resampling iterations. **Results:** PAR demonstrated the highest discriminative performance for 12-month overall survival (OS), with an area under the curve of 0.853. Using an optimal cutoff value of 79.459, patients with elevated PAR experienced significantly shorter OS (median, 6.2 vs. 16.7 months; *p* < 0.001) and progression-free survival (PFS) (median, 5.9 vs. 12.3 months; *p* < 0.001). In baseline-adjusted multivariable analyses, elevated pretreatment PAR remained independently associated with inferior OS (hazard ratio [HR], 3.287; 95% confidence interval [CI], 2.196–4.921; *p* < 0.001) and PFS (HR, 3.791; 95% CI, 2.501–5.749; *p* < 0.001). These findings were supported by sensitivity analyses and bootstrap internal validation. **Conclusions:** Pretreatment PAR was independently associated with survival outcomes and demonstrated favorable discriminative performance relative to other inflammatory biomarkers. PAR may represent an accessible biomarker reflecting tumor–host interactions in IDH-wildtype grade 4 diffuse glioma. The proposed PAR cutoff should be considered exploratory and requires external validation before routine clinical application.

## 1. Introduction

Isocitrate dehydrogenase (IDH)-wildtype grade 4 diffuse glioma is one of the most biologically complex and clinically devastating malignancies in adult oncology, characterized by profound intratumoral heterogeneity, adaptive treatment resistance, and persistently poor survival despite contemporary multimodal therapy [1,2,3]. Contemporary management based on maximal safe resection followed by radiotherapy and temozolomide provides only modest survival gains, while therapeutic strategies that have transformed outcomes in other solid tumors, including immune checkpoint inhibition, have yielded limited benefit in glioblastoma [4,5]. These therapeutic limitations largely reflect the biological complexity of the disease, including an immunologically cold tumor microenvironment and extensive interactions among tumor, vascular, stromal, and immune compartments [6]. Current prognostic models rely primarily on established clinicopathologic and molecular characteristics, including age, performance status, extent of resection, and O6-methylguanine-DNA methyltransferase (MGMT) promoter methylation status [7]. Although these variables provide important prognostic information, they do not fully account for the marked interpatient heterogeneity observed in clinical outcomes. Increasing evidence suggests that survival is influenced not only by intrinsic tumor characteristics but also by systemic host-related biological processes, creating growing interest in biomarkers capable of integrating both tumor- and host-derived determinants of prognosis.

Among these systemic determinants, inflammation, immune regulation, coagulation, metabolic stress, and nutritional status have emerged as important contributors to disease progression and treatment resistance [8,9,10]. Peripheral blood-based biomarkers provide a practical means of capturing these biological processes and have therefore attracted increasing attention in oncology [11]. In diffuse gliomas, inflammatory and immune-nutritional indices, such as the neutrophil-to-lymphocyte ratio (NLR) [12], systemic immune-inflammation index (SII) [13], systemic inflammation response index (SIRI) [14], and pan-immune inflammation value (PIV) [15], have demonstrated prognostic relevance. More broadly, composite biomarkers incorporating inflammatory, metabolic, or nutritional components, such as the C-reactive protein-to-albumin ratio (CAR) [16] and lactate dehydrogenase-to-albumin ratio (LAR) [17], have also been associated with clinical outcomes across diverse solid malignancies. Collectively, these biomarkers reflect complementary dimensions of systemic cancer biology, although none fully captures the complexity of tumor–host interactions that may influence clinical outcomes.

Among the emerging host-response biomarkers, the platelet-to-albumin ratio (PAR) is of particular interest because it integrates two biologically distinct yet interconnected dimensions of cancer progression. Platelets are increasingly recognized as active participants in tumor biology through their roles in angiogenesis, endothelial remodeling, immune modulation, cancer-associated thrombosis, thromboinflammatory signaling and tumor progression [18,19,20]. Conversely, albumin reflects not only nutritional status but also broader aspects of systemic inflammation and physiological reserve, both of which are increasingly recognized as determinants of cancer outcomes [21,22]. By combining these complementary components, PAR may provide a more integrated reflection of tumor–host interactions than biomarkers focused on individual biological domains. Although PAR has demonstrated prognostic value in several solid malignancies [23,24], to our knowledge, its prognostic significance has not previously been investigated in diffuse gliomas, including WHO 2021-defined IDH-wildtype grade 4 diffuse glioma.

Accordingly, the present study was designed to address this unmet knowledge gap by evaluating the prognostic significance of pretreatment PAR within a molecularly defined WHO 2021 cohort and by comparing its performance with established inflammatory biomarkers using comprehensive internal validation strategies.

## 2. Materials and Methods

### 2.1. Study Design and Cohort Assembly

This retrospective single-center cohort study included consecutive patients with histopathologically confirmed diffuse glioma who fulfilled the WHO 2021 diagnostic criteria for IDH-wildtype grade 4 diffuse glioma and were treated at the University of Health Sciences Antalya Training and Research Hospital between January 2017 and July 2025. Because the study period spanned both the pre- and post-WHO 2021 eras, patients diagnosed before publication of the WHO 2021 Classification were retrospectively identified from the institutional pathology database and included only if the available histopathologic and molecular data were consistent with the diagnostic criteria for WHO 2021 IDH-wildtype grade 4 diffuse glioma.

**Figure 1:**
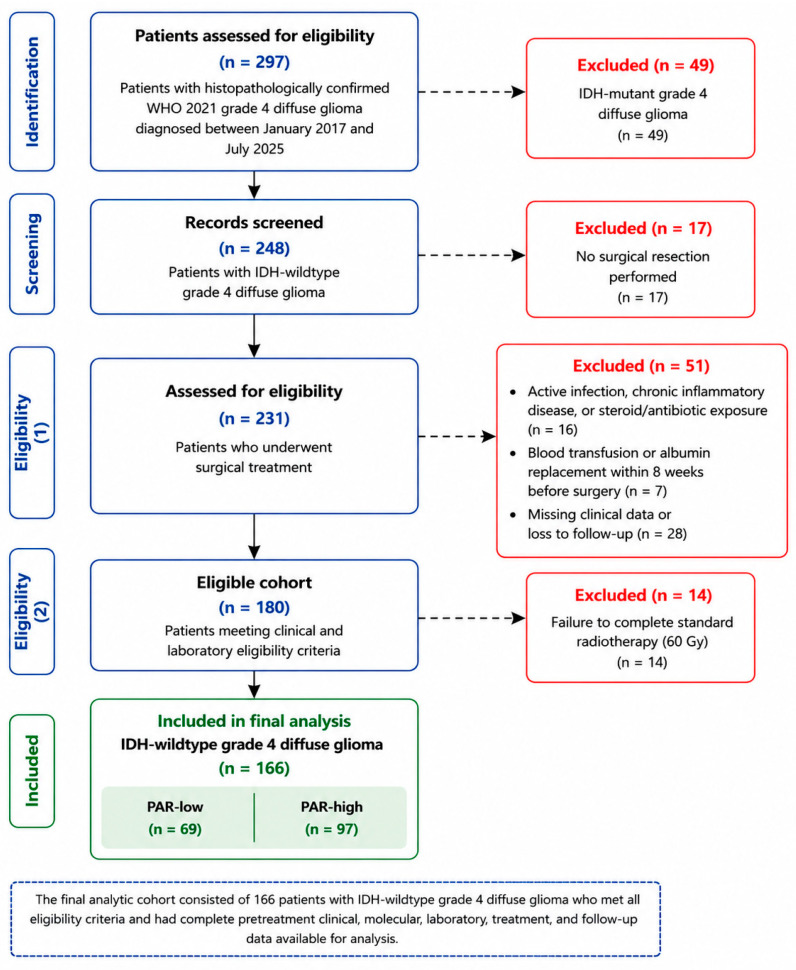
Flow diagram of patient selection and cohort assembly.

A total of 297 patients was screened for their eligibility. After the exclusion of patients with IDH-mutant tumors, those who did not undergo surgical resection or complete standard radiotherapy, individuals with conditions potentially affecting systemic inflammatory biomarkers, and patients with incomplete data or inadequate follow-up, 166 patients were included in the final analysis. The patient selection process is illustrated in Figure 1. The predefined eligibility criteria were intended to establish a clinically homogeneous cohort managed according to a contemporary standard-of-care, thereby reducing treatment-related heterogeneity and facilitating the interpretation of the prognostic associations evaluated in the present study.

Clinical, laboratory, molecular, treatment, and survival data were retrospectively retrieved from the institutional electronic medical records. The collected variables included demographic characteristics, smoking history, Eastern Cooperative Oncology Group (ECOG) performance status, comorbidity status, tumor size, tumor localization, multifocal disease status, extent of surgical resection, MGMT promoter methylation status, adjuvant treatment information, radiologic treatment response, and survival outcomes. Peripheral blood samples used for inflammatory biomarker assessment were obtained as part of the routine preoperative evaluation within seven days before surgical intervention and before any operative procedure or other perioperative intervention.

IDH mutation status was available for all included patients, as the confirmation of IDH-wildtype status constituted an eligibility criterion. MGMT promoter methylation results were available for 86 of the 166 patients (51.8%), whereas the remaining 80 patients (48.2%) had unavailable molecular results. MGMT promoter methylation testing was performed according to routine institutional clinical practice and was therefore unavailable for a proportion of patients, particularly during the earlier years of the study period when routine molecular testing was not consistently available because of technical and financial limitations. Accordingly, molecular characterization was uniform for IDH status but not for MGMT promoter methylation across the study period.

The study protocol was approved by the Institutional Review Board of the University of Health Sciences Antalya Training and Research Hospital (Approval No: 2026-26; Decision No: 1/7; 8 January 2026) and conducted in accordance with the Declaration of Helsinki.

### 2.2. Treatment Protocol and Follow-Up

All patients underwent maximal safe surgical resection according to the institutional multidisciplinary neuro-oncology practice and subsequently received standard postoperative radiotherapy consisting of 60 Gy delivered in 30 fractions. Concurrent temozolomide was administered when clinically appropriate, considering the patient’s performance status, comorbidities, and overall medical suitability. Patients considered eligible for systemic treatment subsequently received adjuvant temozolomide according to the Stupp protocol. Adjuvant treatment was generally planned for six cycles; however, treatment could be extended to up to 12 cycles in selected patients with favorable tolerance and at the discretion of the treating neuro-oncology team.

Treatment response and disease progression were assessed using the Response Assessment in Neuro-Oncology (RANO) 2.0 criteria [25]. Radiologic response was categorized as any response (complete, partial, or minor response) or no response (stable or progressive disease). Postoperative magnetic resonance imaging (MRI) was routinely performed within 48–72 h after surgery. Following the completion of treatment, the patients underwent clinical and radiologic follow-up at regular intervals, generally every three months or earlier when clinically indicated. Progression-free survival events were defined according to the RANO 2.0 progression criteria, and survival status was determined using institutional records and follow-up evaluations.

### 2.3. Calculation of Inflammatory Biomarkers

The primary biomarker of interest was the PAR, which was calculated by dividing the platelet count by the serum albumin concentration [23]. For comparative analyses, additional inflammatory biomarkers were calculated, including NLR, SII, SIRI, PIV, CAR, and LAR. NLR was calculated as neutrophils divided by lymphocytes [12]; SII as platelet count × neutrophils/lymphocytes [13]; SIRI as neutrophils × monocytes/lymphocytes [14]; PIV as neutrophils × platelets × monocytes/lymphocytes [15]; CAR as C-reactive protein divided by albumin [16]; and LAR as lactate dehydrogenase divided by albumin [17].

### 2.4. Study Endpoints

The primary endpoint of this study was overall survival (OS). The secondary endpoints included progression-free survival (PFS), the discriminative performance of pretreatment inflammatory biomarkers for survival outcomes, and the prognostic value of PAR in adjusted survival analyses.

OS was defined as the interval between the date of surgical resection and death from any cause or the last follow-up. PFS was defined as the interval between the date of surgical resection and radiologic or clinical disease progression according to the RANO 2.0 criteria [25], death from any cause, or last follow-up, whichever occurred first. Patients without documented progression or death were censored on the date of their last clinical or radiologic assessment.

### 2.5. Statistical Analysis

Statistical analyses were performed using IBM SPSS Statistics for Windows, version 27.0 (IBM Corp., Armonk, NY, USA), and R software version 4.4.1 (R Foundation for Statistical Computing, Vienna, Austria). Continuous variables were assessed for normality using the Shapiro–Wilk test and are presented as median (interquartile range [IQR]) or frequency (percentage), as appropriate. Comparisons between groups were performed using the Mann–Whitney U test for continuous variables and the χ^2^ test or Fisher’s exact test for categorical variables.

The discriminative performance of the pretreatment inflammatory biomarkers was evaluated using receiver operating characteristic (ROC) analyses. The optimal PAR cutoff was determined from the 12-month OS ROC curve using the Youden index and subsequently applied across all discrimination and survival analyses. To account for censoring and evaluating temporal changes in prognostic performance, inverse probability of censoring weighting (IPCW)-adjusted time-dependent ROC analyses were performed at predefined landmark time points (6, 12, 18, and 24 months) for both OS and PFS. Pairwise comparisons of AUC values were performed using the DeLong method, with PAR serving as the reference biomarker.

The median follow-up duration was estimated using the reverse Kaplan–Meier method. Survival outcomes were evaluated using Kaplan–Meier analyses and Cox proportional hazards regression models. Exploratory interaction analyses were performed by incorporating multiplicative interaction terms between PAR status and clinically relevant subgroup variables into the baseline-adjusted Cox models.

To evaluate the independent prognostic significance of pretreatment PAR, multivariable Cox regression analyses were performed using a predefined baseline-adjusted modeling strategy. Model 1 included baseline clinicopathologic and molecular variables selected a priori based on established clinical relevance, including age, ECOG performance status, multifocal disease, tumor size, extent of surgical resection, and MGMT promoter methylation status. Model 2 additionally incorporated pretreatment PAR status to assess its incremental prognostic value beyond established baseline prognostic factors. MGMT promoter methylation status was modeled as a three-level categorical variable (methylated, unmethylated, and unknown), with the unmethylated group serving as the reference category. Treatment-related post-baseline variables, including the receipt of adjuvant chemotherapy and radiologic response to adjuvant therapy, were not included in the primary multivariable models. Hazard ratios (HRs) and corresponding 95% confidence intervals (CIs) were reported.

Sensitivity analyses were performed by incorporating continuous PAR values into the multivariable models as a per-10-unit increase variable. Potential multicollinearity among variables included in the multivariable analyses was assessed using variance inflation factors (VIFs). The proportional hazards assumption was evaluated for each Cox regression model using Schoenfeld residual-based diagnostics. Internal validation was performed using 1000 bootstrap resampling iterations applied to the final multivariable models. All statistical tests were two-sided, and *p* values <0.05 were considered statistically significant.

### 2.6. Artificial Intelligence Statement

During manuscript preparation, ChatGPT-5.5 (OpenAI) was used exclusively to assist with language editing, stylistic refinement, and improvement in readability. The AI-generated output was critically reviewed, revised, and verified by the authors. All study conception, methodology, statistical analyses, data interpretation, and final editorial decisions were performed exclusively by the authors, who assume full responsibility for the integrity and accuracy of the work.

## 3. Results

### 3.1. Baseline Clinicopathologic Characteristics

The final analytic cohort comprised 166 patients with IDH-wildtype grade 4 diffuse glioma. The median age at diagnosis was 60.0 years (IQR, 53.0–67.0), and 92 patients (55.4%) were men. ECOG performance status was zero to one in 123 patients (74.1%) and ≥2 in 43 patients (25.9%). Multifocal disease was present in 41 patients (24.7%), and gross total resection was achieved in 77 (46.4%). The median tumor size was 44.0 mm (IQR, 35.0–55.0 mm). Additional baseline clinicopathological characteristics are summarized in Table 1.

Based on the predefined PAR cutoff value of 79.459, 69 patients (41.6%) were classified as PAR-low and 97 patients (58.4%) as PAR-high.

Compared with the PAR-low group, patients in the PAR-high group more frequently exhibited ECOG performance status ≥ 2 (*p* < 0.001), multifocal disease (*p* = 0.017), and larger tumor size at diagnosis (*p* = 0.027). No significant differences were observed between the PAR-defined groups with respect to age, sex, smoking history, comorbidity status, tumor lateralization, tumor origin, extent of surgical resection, or history of epileptic seizure at diagnosis (all *p* > 0.05).

PAR-high patients also demonstrated higher frequencies of elevated NLR, SII, SIRI, PIV, CAR, and LAR values (all *p* < 0.001). Detailed comparisons of the baseline clinicopathological and inflammatory characteristics according to PAR status are presented in Table 1.

### 3.2. Diagnostic and Discriminative Performance of Pretreatment Inflammatory Biomarkers

**Figure 2:**
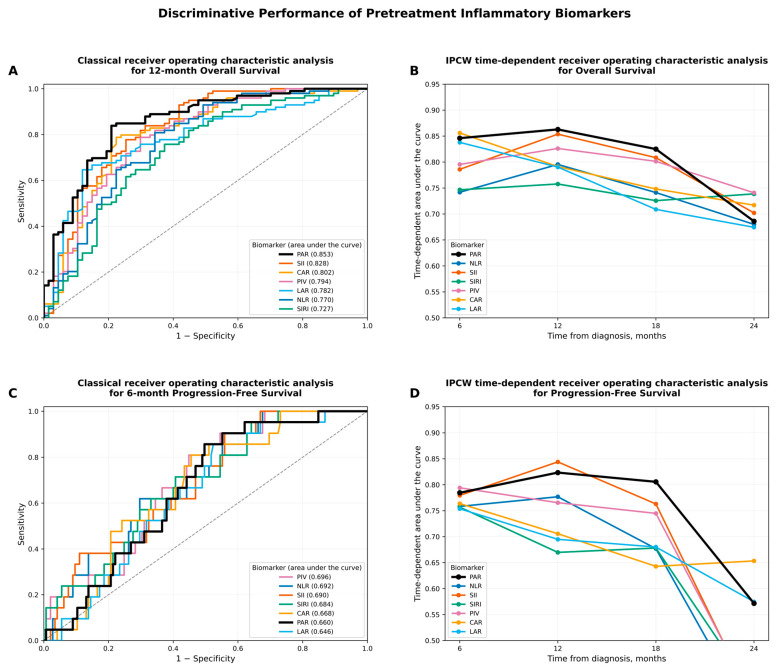
Discriminative performance of pretreatment inflammatory biomarkers for survival outcomes in IDH-Wildtype Grade 4 Diffuse Glioma. (**A**) 12 month overall survival; (**B**) inverse probability of censoring weighting (IPCW)-adjusted time-dependent ROC analysis for overall survival; (**C**) classical ROC analysis for 6-month progression-free survival; (**D**) IPCW-adjusted time-dependent ROC analysis for progression-free survival. Area under the curve values for classical ROC analyses are presented in the corresponding legends. Abbreviations: CAR, C-reactive protein-to-albumin ratio; IPCW, inverse probability of censoring weighting; LAR, lactate dehydrogenase-to-albumin ratio; NLR, neutrophil-to-lymphocyte ratio; PAR, platelet-to-albumin ratio; PIV, pan-immune-inflammation value; ROC, receiver operating characteristic; SII, systemic immune-inflammation index; SIRI, systemic inflammation response index.

**Figure 3:**
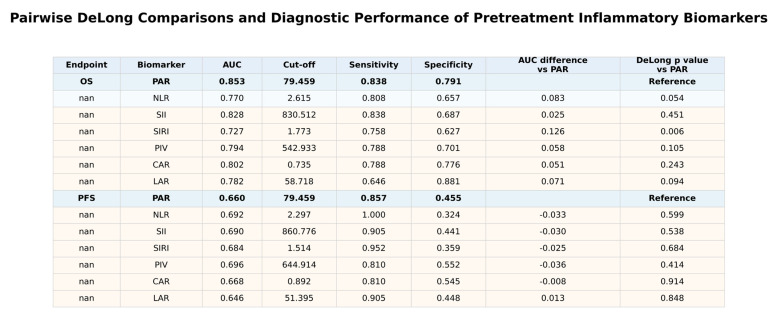
Pairwise delong comparisons and diagnostic performance of pretreatment inflammatory biomarkers. The platelet-to-albumin ratio (PAR) cutoff was derived from the 12-month overall survival receiver operating characteristic analysis and was subsequently applied across all survival analyses. Pairwise comparisons of the area under the curve values were performed using the DeLong method, with PAR as the reference biomarker. Abbreviations: AUC, area under the curve; CAR, C-reactive protein-to-albumin ratio; LAR, lactate dehydrogenase-to-albumin ratio; NLR, neutrophil-to-lymphocyte ratio; PAR, platelet-to-albumin ratio; PFS, progression-free survival; PIV, pan-immune-inflammation value; SII, systemic immune-inflammation index; SIRI, systemic inflammation response index.

In the classical ROC analysis for 12-month overall survival, pretreatment PAR achieved the highest discriminative performance among the evaluated inflammatory biomarkers, with an AUC of 0.853 (Figure 2A). The corresponding AUC values were 0.828 for SII, 0.802 for CAR, 0.794 for PIV, 0.782 for LAR, 0.770 for NLR, and 0.727 for SIRI. The optimal PAR cutoff value identified using the Youden index was 79.459, corresponding to a sensitivity of 83.8% and specificity of 79.1% (Figure 3).

In IPCW-adjusted time-dependent ROC analyses for overall survival, PAR consistently demonstrated among the highest AUC values across evaluated landmark time points, including 0.863 at 12 months and 0.825 at 18 months (Figure 2B).

Pairwise DeLong analyses for 12-month overall survival demonstrated superior discriminative performance for PAR compared with SIRI (AUC difference, 0.126; *p* = 0.006), whereas no statistically significant differences were observed between PAR and the other inflammatory biomarkers (Figure 3).

The discriminative performance was generally lower for progression-free survival than for overall survival (Figure 2C). In the classical ROC analysis for 6-month progression-free survival, PIV demonstrated the numerically highest AUC (0.696), followed by NLR (0.692), SII (0.690), SIRI (0.684), CAR (0.668), PAR (0.660), and LAR (0.646). Using the predefined overall survival-derived cutoff value, the PAR demonstrated a sensitivity of 85.7% and a specificity of 45.5% for 6-month progression-free survival (Figure 3).

In IPCW-adjusted time-dependent ROC analyses for progression-free survival, PAR yielded AUC values of 0.783, 0.824, 0.806, and 0.573 at 6, 12, 18, and 24 months, respectively (Figure 2D). Pairwise DeLong analyses demonstrated no statistically significant differences between PAR and the remaining inflammatory biomarkers for progression-free survival (all *p* > 0.05) (Figure 3).

### 3.3. Survival Analyses According to Pretreatment Platelet-to-Albumin Ratio

At the data cutoff, disease progression was observed in 155 patients (93.4%), whereas 139 patients (83.7%) had died. The median follow-up, estimated using the reverse Kaplan–Meier method, was 36.4 months (95% CI, 26.1–not reached). The median OS for the cohort was 10.8 months (95% CI, 8.9–12.7), and the median PFS was 8.3 months (95% CI, 6.5–10.1).

Using the predefined PAR cutoff value of 79.459, patients were stratified into PAR-low and PAR-high groups for all survival analyses.

**Figure 4:**
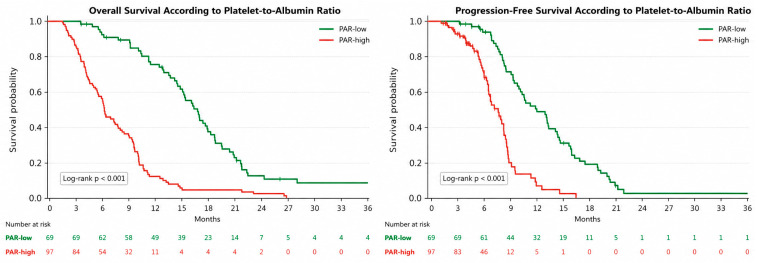
Kaplan–Meier survival analyses according to pretreatment platelet-to-albumin ratio. Abbreviations: PAR, platelet-to-albumin ratio. Patients were stratified using a predefined platelet-to-albumin ratio (PAR) cutoff value. The tick marks indicate censored observations.

Kaplan–Meier analyses demonstrated significantly inferior overall and progression-free survival in patients with elevated pretreatment PAR levels. The median OS was 16.7 months (95% CI, 14.7–17.8) in the PAR-low group compared with 6.2 months (95% CI, 5.5–7.7) in the PAR-high group (log-rank *p* < 0.001) (Figure 4). The median PFS was 12.3 months (95% CI, 10.2–13.6) in the PAR-low group and 5.9 months (95% CI, 4.8–8.5) in the PAR-high group (log-rank *p* < 0.001) (Figure 4).

**Figure 5:**
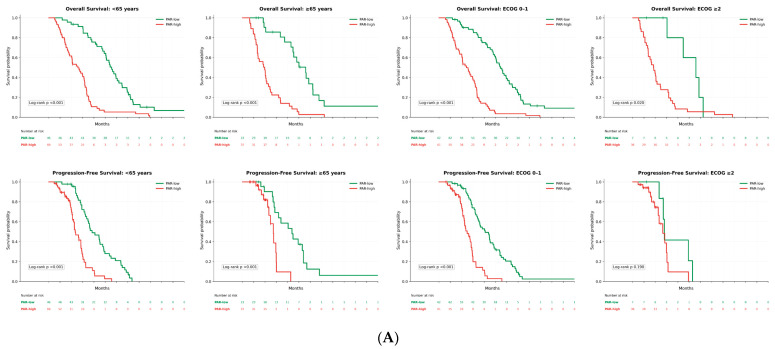

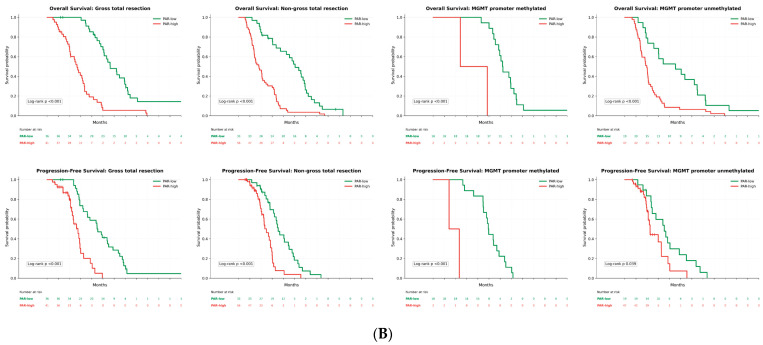
(**A**) Kaplan–meier survival analyses according to pretreatment platelet-to-albumin ratio across clinical subgroups. Abbreviations: ECOG, Eastern Cooperative Oncology Group; PAR, platelet-to-albumin ratio. Kaplan–Meier analyses were stratified according to age and ECOG performance status using the predefined platelet-to-albumin ratio (PAR) cutoff value. The tick marks indicate the censored observations. (**B**) Kaplan–Meier survival analyses according to pretreatment platelet-to-albumin ratio across surgical and molecular subgroups. Abbreviations: GTR, gross total resection; MGMT, O6-methylguanine-DNA methyltransferase; PAR, platelet-to-albumin ratio. Kaplan–Meier analyses were stratified according to the extent of surgical resection and MGMT promoter methylation status using the predefined platelet-to-albumin ratio (PAR) cutoff value. The tick marks indicate the censored observations.

Exploratory subgroup analyses demonstrated generally consistent survival separation between the PAR-defined groups across age, ECOG performance status, extent of surgical resection, and MGMT promoter methylation subgroups for both OS and PFS (Figure 5A,B). Formal interaction testing did not identify statistically significant heterogeneity in the association between PAR and survival outcomes across these evaluated subgroups.

### 3.4. Prognostic Factors Associated with Survival Outcomes

In univariable Cox regression analyses, ECOG performance status ≥ 2, multifocal disease, absence of gross total resection, lack of adjuvant chemotherapy, unfavorable response to adjuvant therapy, and elevated pretreatment PAR were significantly associated with inferior OS (Table 2). Elevated pretreatment PAR demonstrated one of the strongest univariable associations with OS (HR, 3.90; 95% CI, 2.74–5.56; *p* < 0.001). Similar associations were observed for PFS, whereas ECOG performance status showed borderline significance (HR, 1.63; 95% CI, 0.98–2.72; *p* = 0.059). MGMT promoter methylation status was significantly associated with OS in the univariable analysis (HR, 1.44; 95% CI, 1.02–2.04; *p* = 0.037), whereas no significant association was observed for PFS.

Multivariable Cox regression analyses were subsequently reconstructed using a predefined baseline-adjusted modeling strategy (Table 3). Model 1 included age, ECOG performance status, multifocal disease, tumor size, extent of surgical resection, and MGMT promoter methylation status as baseline clinicopathologic and molecular covariates. Model 2 additionally incorporated pretreatment PAR status.

In the baseline-adjusted multivariable analyses, elevated pretreatment PAR remained independently associated with both survival endpoints. In Model 2, PAR-high status was associated with inferior OS (HR, 3.287; 95% CI, 2.196–4.921; *p* < 0.001) and inferior PFS (HR, 3.791; 95% CI, 2.501–5.749; *p* < 0.001). Gross total resection remained independently associated with improved OS (HR, 0.681; 95% CI, 0.481–0.963; *p* = 0.030), whereas ECOG performance status ≥ 2 remained independently associated with shorter PFS (HR, 1.504; 95% CI, 1.022–2.213; *p* = 0.038).

MGMT promoter methylation status was analyzed as a three-level categorical variable (methylated, unmethylated, and unknown). Compared with the unmethylated group, MGMT methylation was associated with improved OS and PFS in Model 1; however, these associations did not remain statistically significant after the incorporation of PAR into Model 2. Patients with unknown MGMT promoter methylation status were retained in the multivariable analyses and showed no statistically significant differences in OS or PFS relative to the unmethylated reference group.

VIF values ranged from 1.086 to 1.376 across all variables included in the final baseline-adjusted multivariable models, indicating no evidence of substantial multicollinearity. Schoenfeld residual-based diagnostics demonstrated evidence of non-proportionality in the overall survival models and in the baseline progression-free survival model, whereas the final progression-free survival model including PAR satisfied the proportional hazards assumption. Importantly, no statistically significant violation of the proportional hazards assumption was observed for PAR in the final overall survival or progression-free survival models. Accordingly, Cox-derived hazard ratios should be interpreted as average associations over the observed follow-up period.

### 3.5. Sensitivity and Internal Validation Analyses

To further evaluate the robustness of the primary findings, sensitivity analyses were performed using continuous PAR values incorporated into the multivariable models as a per 10-unit increase variable. Continuous PAR remained independently associated with shorter PFS (HR, 1.22; 95% CI, 1.04–1.43; *p* = 0.014) and demonstrated a borderline association with OS (HR, 1.14; 95% CI, 0.99–1.30; *p* = 0.060) (Table 4A).

No substantial multicollinearity was identified among the variables included in the continuous PAR models, with all variance inflation factors remaining below 2.

Internal validation using 1000 bootstrap resampling iterations demonstrated stable PAR-associated hazard ratio estimates across repeated resampled datasets. The median bootstrap hazard ratios were 1.75 for OS and 2.27 for PFS, with hazard ratios remaining greater than one in 82.2% and 88.4% of bootstrap iterations, respectively (Table 4B).

## 4. Discussion

The present study demonstrates that pretreatment PAR was independently associated with both OS and PFS in patients with IDH-wildtype grade 4 diffuse glioma after adjustment for established baseline clinicopathologic and molecular prognostic factors. Among the inflammatory biomarkers evaluated, PAR exhibited the highest numerical discriminative performance for 12-month overall survival. This numerical advantage, however, translated into statistically significant superiority only over SIRI, whereas no significant differences were observed relative to NLR, SII, PIV, CAR, or LAR. Rather than supporting broad superiority over existing inflammatory biomarkers, these findings position PAR among the highest-performing biomarkers evaluated and suggest that it may serve as a robust complementary prognostic indicator within a multimarker risk assessment framework. The observed associations were consistent across clinically relevant subgroup analyses and were further supported by bootstrap internal validation. Sensitivity analyses modeling PAR as a continuous variable demonstrated directionally consistent associations with both survival endpoints, although statistical significance was retained only for progression-free survival, whereas the association with overall survival remained borderline. Collectively, these findings suggest that PAR may reflect a biologically relevant dimension of the tumor–host interface, integrating platelet-associated thromboinflammatory activity with systemic physiological reserve. Nevertheless, PAR is unlikely to reflect a purely isolated biological process. Because platelet counts and serum albumin levels are influenced not only by tumor-associated inflammatory signaling but also by disease burden and the patient’s overall physiological condition, PAR may partly reflect systemic disease burden and host physiological reserve in addition to tumor–host interactions. The persistence of its prognostic association after comprehensive adjustment for established baseline clinicopathologic and molecular prognostic factors nevertheless suggests that PAR provides complementary prognostic information beyond these conventional determinants rather than merely acting as a surrogate of underlying disease burden.

The biological plausibility of these findings is supported by accumulating evidence implicating platelets in glioma progression and treatment resistance [26,27]. Beyond their canonical role in hemostasis, platelets actively participate in tumor biology through the release of growth factors, cytokines, and proangiogenic mediators that influence endothelial remodeling, vascular permeability, and immune regulation [28]. This biology may be particularly relevant in IDH-wildtype grade 4 diffuse glioma, a disease characterized by pronounced angiogenesis, hypoxia, necrosis, and a highly dynamic tumor microenvironment [6,19]. Previous clinical studies have associated preoperative thrombocytosis with inferior outcomes in glioblastoma, while platelet-related molecular signatures have been linked to hypoxia-associated pathways and mesenchymal tumor phenotypes [29]. Experimental evidence further suggests that platelet-derived signaling may promote glioma stem cell maintenance, proliferation, and invasive behavior, providing a mechanistic framework through which elevated PAR may reflect a more aggressive tumor state [30].

This platelet-centered biology may be particularly relevant in glioblastoma, a malignancy characterized by profound interactions between tumor progression, coagulation, and systemic inflammation [31]. Glioblastoma is among the solid tumors most strongly associated with cancer-related thrombosis, highlighting the close relationship between vascular dysfunction, thromboinflammatory activation, and disease aggressiveness [32]. Within this framework, an elevated platelet component of PAR may reflect more than a nonspecific hematologic alteration; rather, it may be indicative of a systemic host–tumor state enriched with platelet activation, endothelial remodeling, and hypoxia-associated signaling. This interpretation is further supported by prior observations linking increased platelet counts during treatment to inferior survival outcomes in glioblastoma [33,34], suggesting that platelet-related biomarkers may serve as dynamic surrogates of aggressive disease biology rather than merely reflecting treatment effects or marrow reserve.

The prognostic relevance of PAR is unlikely to be driven solely by platelet biology. The albumin component may provide complementary information regarding host physiologic reserve and the systemic consequences of cancer-associated inflammation. As a negative acute-phase reactant, albumin levels decline in the setting of sustained inflammatory signaling and may reflect a broader state of immunometabolic vulnerability [21,35]. In patients with aggressive malignancies, hypoalbuminemia has been associated with chronic inflammatory burden, catabolic stress, impaired nutritional status, reduced treatment tolerance, and progressive physiologic decline [36]. Consequently, PAR integrates two biologically interconnected dimensions of disease: platelet-associated protumoral and thromboinflammatory activity in the numerator, and host reserve in the denominator [23]. This integrated structure may partly explain why PAR demonstrated favorable prognostic performance relative to biomarkers that primarily reflect leukocyte composition or individual inflammatory pathways.

The comparative biomarker findings of this study further support the potential biological relevance of PAR. Although NLR, SII, SIRI, PIV, CAR, and LAR were each associated with survival outcomes in univariable analyses, PAR demonstrated one of the strongest discriminative performances for overall survival and retained independent prognostic significance after multivariable adjustment. Previous studies on glioblastoma have highlighted the prognostic value of systemic inflammatory and immune-nutritional biomarkers, including NLR [12] and composite indices such as SII [13], SIRI [14], PIV [15], and CAR [37], suggesting that peripheral host-response markers may provide information beyond conventional clinicopathologic variables. Beyond prognostic stratification, emerging evidence also supports the potential role of blood-based biomarkers in addressing clinically challenging scenarios, such as differentiating pseudoprogression from true tumor progression after standard multimodal therapy [38]. However, many existing indices primarily reflect leukocyte composition and individual inflammatory pathways. In contrast, PAR may integrate distinct yet complementary dimensions of disease biology by simultaneously capturing platelet-associated tumor-promoting activity and the host’s physiological reserve. This integrative framework may account for its favorable prognostic performance and supports its potential role as a clinically accessible biomarker of the tumor–host interface.

The stronger discriminative performance of PAR for OS than for PFS is biologically plausible given the distinct biological processes reflected by these endpoints. Whereas PFS primarily reflects the timing of disease progression, OS represents the cumulative consequences of tumor biology, systemic host condition, treatment tolerance, and subsequent therapeutic interventions. By integrating platelet-associated thromboinflammatory activity with host physiological reserve through its albumin component, PAR may preferentially capture biological processes that influence long-term survival rather than disease progression alone. This interpretation is consistent with the favorable prognostic performance of PAR observed across multiple predefined landmark time points in the IPCW-adjusted time-dependent ROC analyses.

Several aspects of the present findings support the potential clinical relevance of PAR. Notably, the PAR threshold was derived from the 12-month overall survival ROC analysis and subsequently applied across all survival analyses to maintain analytical consistency. However, because this threshold was both derived and evaluated within the same cohort, it should be regarded as an exploratory, cohort-derived threshold that requires validation in independent external cohorts before broader clinical application. Furthermore, the prognostic association was not confined to the overall cohort but remained generally consistent across clinically meaningful subgroups defined by age, performance status, extent of resection, and MGMT promoter methylation status. Formal interaction analyses did not demonstrate statistically significant heterogeneity in the association between PAR and survival outcomes across these predefined subgroups. Nevertheless, because of the limited sample size within individual strata, these subgroup analyses should be interpreted as exploratory assessments of the consistency of the overall findings rather than as definitive evidence of effect modification. The persistence of these associations after adjustment for established baseline prognostic factors, together with broadly supportive findings from the bootstrap internal validation analyses, suggests that PAR may capture prognostic information beyond that conveyed by routine baseline laboratory measurements alone. Although bootstrap analyses generally supported the observed prognostic associations, these findings were not reproduced uniformly across all resampling iterations and should therefore be interpreted as providing supportive rather than definitive evidence of internal model stability. Nevertheless, these findings should be regarded as hypothesis-generating rather than practice-changing and warrant external validation before broader clinical implementation.

The potential clinical utility of the PAR stems from its prognostic performance and practicality. As a biomarker derived from routinely available laboratory parameters, PAR can be obtained before surgery or chemoradiotherapy without additional molecular testing, specialized assays, or advanced imaging infrastructure. This accessibility may be particularly relevant in real-world neuro-oncology practice, where the availability of sophisticated prognostic platforms can vary substantially across institutions and health care systems. Importantly, the PAR should not be viewed as a substitute for established prognostic determinants, such as age, performance status, extent of resection, MGMT promoter methylation, or treatment-related factors. Rather, its potential value may lie in complementing these variables by providing a systemic host-response dimension that is not fully captured by tumor-centered biomarkers alone. Whether incorporation of PAR into established prognostic models provides incremental predictive value or meaningfully improves clinical risk stratification and therapeutic decision-making remains to be established through prospective external validation studies.

This study had some limitations. Its retrospective, single-center design and predefined eligibility criteria introduce the possibility of selection bias, unmeasured confounding, and limited generalizability. In particular, restricting the cohort to patients treated according to a contemporary standard-of-care may have excluded individuals with alternative treatment pathways or poorer clinical status. However, this design was intentionally adopted to establish a clinically homogeneous study population and thereby reduce treatment-related heterogeneity for the primary prognostic analyses. Although the multivariable analyses incorporated major baseline clinicopathologic and molecular prognostic factors, residual confounding from unmeasured variables cannot be excluded. Furthermore, although MGMT promoter methylation status was incorporated into the multivariable models using a three-level categorical variable to retain patients with unavailable molecular results, incomplete molecular characterization remained an inherent limitation of this retrospective cohort. In addition, the optimal PAR threshold was derived and evaluated within the same study cohort. Although this approach is widely used in exploratory biomarker studies, it may introduce optimism bias and overestimate discriminatory performance. Accordingly, the proposed threshold should be considered hypothesis-generating and requires validation in independent external cohorts before broader clinical implementation. Several clinically relevant factors that may influence platelet counts, serum albumin concentrations, and systemic inflammatory biomarkers—including corticosteroid exposure, infection, thromboembolic events, antiplatelet or anticoagulant therapy, perioperative inflammatory complications, and longitudinal changes in platelet or albumin levels—could not be comprehensively captured because of the retrospective nature of the study. Finally, PAR was evaluated as a peripheral blood-based surrogate biomarker, and direct mechanistic validation through tumor-based analyses, platelet activation profiling, cytokine characterization, or spatial interrogation of immune–vascular interactions was beyond the scope of this study.

## 5. Conclusions

In conclusion, pretreatment PAR was independently associated with both OS and PFS after adjustment for established baseline clinicopathologic and molecular prognostic factors in patients with IDH-wildtype grade 4 diffuse glioma. These findings suggest that PAR may serve as a clinically accessible surrogate marker of biological processes occurring at the tumor–host interface, integrating platelet-associated thromboinflammatory processes with diminished systemic physiological reserve. Given its accessibility and favorable discriminative performance, PAR warrants further evaluation as a clinically accessible prognostic biomarker through external multicenter validation and translational studies integrating peripheral blood biomarkers with the molecular, vascular, and immune features of the glioma microenvironment.

## Figures and Tables

**Table 1:** Baseline clinicopathologic characteristics according to pretreatment platelet-to-albumin ratio (*n* = 166).

Variable		Overall, *n* (%)	PAR-Low, *n* (%)	PAR-High, *n* (%)	*p*
Age, years		60.0 (53.0–67.0)	59.0 (53.0–66.0)	62.0 (53.0–70.0)	0.245
Sex	Male	92 (55.4)	37 (53.6)	55 (56.7)	0.814
	Female	74 (44.6)	32 (46.4)	42 (43.3)	
Comorbidity	Yes	74 (44.6)	28 (40.6)	46 (47.4)	0.474
	No	92 (55.4)	41 (59.4)	51 (52.6)	
Smoking	Yes	62 (37.3)	24 (34.8)	38 (39.2)	0.679
	No	104 (62.7)	45 (65.2)	59 (60.8)	
ECOG performance status	0	18 (10.8)	12 (17.4)	6 (6.2)	<0.001
	1	105 (63.3)	50 (72.5)	55 (56.7)	
	2	43 (25.9)	7 (10.1)	36 (37.1)	
Tumor lateralization	left	83 (50.0)	34 (49.3)	49 (50.5)	1.000
	right	83 (50.0)	35 (50.7)	48 (49.5)	
The cerebral lobe of tumor origin	parietal	54 (32.5)	17 (24.6)	37 (38.1)	0.220
	frontal	46 (27.7)	20 (29.0)	26 (26.8)	
	temporal	44 (26.5)	23 (33.3)	21 (21.6)	
	other	22 (13.3)	9 (13.0)	13 (13.4)	
Tumor focality	unifocal	125 (75.3)	59 (85.5)	66 (68.0)	0.017
	multifocal	41 (24.7)	10 (14.5)	31 (32.0)	
Tumor size, mm		44.0 (35.0–55.0)	40.5 (31.8–50.5)	45.0 (37.0–55.0)	0.027
Type of surgery	GTR	77 (46.4)	36 (52.2)	41 (42.3)	0.270
	Non-GTR	89 (53.6)	33 (47.8)	56 (57.7)	
Adjuvant chemotherapy	No	35 (21.1)	11 (15.9)	24 (24.7)	<0.001
	Yes	131 (78.9)	58 (84.1)	73 (75.3)	
MGMT promoter methylation status	Unmethylated	66 (39.8)	19 (27.5)	47 (48.5)	<0.001
	Methylated	20 (12.0)	18 (26.1)	2 (2.0)	
	Unknown	80 (48.2)	32 (46.4)	48 (49.5)	
Response to adjuvant therapy	Any response	71 (54.2)	49 (75.4)	22 (33.3)	<0.001
	No response	60 (45.8)	16 (24.6)	44 (66.7)	
Repeat surgery at progression	No	67 (59.3)	31 (52.5)	36 (66.7)	0.182
	Yes	46 (40.7)	28 (47.5)	18 (33.3)	
Receipt of chemotherapy at progression	No	61 (54.0)	26 (44.1)	35 (64.8)	0.043
	Yes	52 (46.0)	33 (55.9)	19 (35.2)	
NLR, high (≥2.61)		102 (61.4)	28 (40.6)	74 (76.3)	<0.001
SII, high (≥830.51)		103 (62.0)	20 (29.0)	83 (85.6)	<0.001
SIRI, high (≥1.77)		99 (59.6)	29 (42.0)	70 (72.2)	<0.001
PIV, high (≥542.93)		97 (58.4)	22 (31.9)	75 (77.3)	<0.001
CAR, high (≥0.73)		93 (56.0)	21 (30.4)	72 (74.2)	<0.001
LAR, high (≥58.71)		71 (42.8)	11 (15.9)	60 (61.9)	<0.001

Abbreviations: CAR, C-reactive protein-to-albumin ratio; ECOG, Eastern Cooperative Oncology Group; GTR, gross total resection; IQR, interquartile range; LAR, lactate dehydrogenase-to-albumin ratio; NLR, neutrophil-to-lymphocyte ratio; PAR, platelet-to-albumin ratio; PIV, pan-immune inflammation value; ROC, receiver operating characteristic; SII, systemic immune-inflammation index; SIRI, systemic inflammation response index. Values are presented as median (IQR) or number (%). Continuous variables were compared using the Mann–Whitney U test, and categorical variables were compared using the χ^2^ test or Fisher’s exact test, as appropriate. Inflammatory biomarkers were dichotomized using 12-month overall survival ROC-derived Youden cutoffs.

**Table 2:** Univariable Cox regression analyses for overall and progression-free survival.

Variable	Overall Survival			Progression-Free Survival		
	HR	95% CI	*p*	HR	95% CI	*p*
Age	1.011	0.997–1.026	0.135	0.993	0.976–1.011	0.444
Male sex	1.043	0.756–1.440	0.798	0.966	0.663–1.409	0.858
ECOG ≥ 2	1.928	1.340–2.773	<0.001	1.634	0.981–2.721	0.059
Right-sided tumor	0.75	0.542–1.037	0.082	0.788	0.544–1.141	0.207
Multifocal disease	1.821	1.255–2.641	0.002	2.111	1.361–3.274	<0.001
Tumor size	1.007	0.996–1.017	0.209	1.007	0.996–1.018	0.232
Gross total resection	0.583	0.421–0.808	0.001	0.63	0.432–0.919	0.016
MGMT methylated	1.444	1.023–2.038	0.037	1.165	0.769–1.765	0.471
Adjuvant chemotherapy	0.138	0.092–0.209	<0.001	0.236	0.105–0.527	<0.001
Favorable response to adjuvant therapy	0.294	0.199–0.433	<0.001	0.211	0.134–0.335	<0.001
Surgery at progression	0.63	0.430–0.925	0.018	0.683	0.465–1.004	0.053
Chemotherapy at progression	0.369	0.248–0.549	<0.001	0.706	0.485–1.027	0.069
PAR-high	3.904	2.740–5.562	<0.001	3.7	2.418–5.663	<0.001
NLR-high	1.103	1.055–1.153	<0.001	1.085	1.029–1.143	0.002
SII-high	1.0	1.000–1.000	<0.001	1.0	1.000–1.001	<0.001
SIRI-high	1.276	1.168–1.394	<0.001	1.272	1.131–1.431	<0.001
PIV-high	1.001	1.001–1.001	<0.001	1.001	1.001–1.001	<0.001
CAR-high	1.226	1.129–1.332	<0.001	1.137	0.998–1.296	0.053
LAR-high	1.02	1.014–1.026	<0.001	1.016	1.007–1.025	<0.001

Abbreviations: CAR, C-reactive protein-to-albumin ratio; CI, confidence interval; ECOG, Eastern Cooperative Oncology Group; GTR, gross total resection; HR, hazard ratio; LAR, lactate dehydrogenase-to-albumin ratio; MGMT, O6-methylguanine-DNA methyltransferase; NLR, neutrophil-to-lymphocyte ratio; PAR, platelet-to-albumin ratio; PFS, progression-free survival; PIV, pan-immune inflammation value; SII, systemic immune-inflammation index; SIRI, systemic inflammation response index. Hazard ratios for overall survival and progression-free survival were estimated using separate univariable Cox proportional hazard regression models. MGMT promoter methylation analyses included only methylated and unmethylated tumors; patients with unknown MGMT methylation status were excluded from the regression analyses.

**Table 3:** Baseline-adjusted multivariable Cox regression models for overall and progression-free survival.

Variable	Overall Survival				Progression-Free Survival			
	Model 1 HR (95% CI)	*p*	Model 2 HR (95% CI)	*p*	Model 1 HR (95% CI)	*p*	Model 2 HR (95% CI)	*p*
Age	1.006 (0.990–1.021)	0.485	1.006 (0.990–1.022)	0.476	1.004 (0.988–1.019)	0.638	1.005 (0.989–1.021)	0.531
ECOG ≥ 2	1.787 (1.232–2.591)	0.002	1.167 (0.791–1.721)	0.436	2.088 (1.426–3.057)	<0.001	1.504 (1.022–2.213)	0.038
Multifocal disease	1.426 (0.948–2.146)	0.088	1.261 (0.832–1.911)	0.275	1.319 (0.876–1.986)	0.184	1.194 (0.790–1.806)	0.400
Tumor size	1.001 (0.990–1.012)	0.915	1.004 (0.992–1.016)	0.549	0.998 (0.988–1.009)	0.723	1.001 (0.990–1.013)	0.815
Gross total resection	0.733 (0.515–1.042)	0.083	0.681 (0.481–0.963)	0.030	0.747 (0.524–1.063)	0.105	0.735 (0.523–1.034)	0.077
MGMT methylated	0.411 (0.245–0.692)	0.001	0.705 (0.403–1.235)	0.222	0.389 (0.229–0.660)	<0.001	0.694 (0.393–1.228)	0.210
MGMT unknown	0.766 (0.538–1.093)	0.142	0.881 (0.617–1.258)	0.487	0.728 (0.512–1.034)	0.076	0.816 (0.576–1.156)	0.252
PAR-high	—	—	3.287 (2.196–4.921)	<0.001	—	—	3.791 (2.501–5.749)	<0.001

Abbreviations: CI, confidence interval; ECOG, Eastern Cooperative Oncology Group; GTR, gross total resection; HR, hazard ratio; MGMT, O6-methylguanine-DNA methyltransferase; PAR, platelet-to-albumin ratio; PFS, progression-free survival. Model 1 included predefined baseline clinicopathologic and molecular covariates. Model 2 additionally incorporated pretreatment platelet-to-albumin ratio status. MGMT promoter methylation status was modeled as a three-level categorical variable, with unmethylated tumors serving as the reference category. Treatment-related post-baseline variables were not included in the primary multivariable models to avoid potential overadjustment and collider bias.

**Table 4:** (**A**) Sensitivity analyses using continuous platelet-to-albumin ratio. (**B**) Bootstrap internal validation of PAR-associated multivariable models.

**(A)**					
**Variable**		**Overall Survival**		**Progression-Free Survival**	
		**HR (95% CI)**	***p***	**HR (95% CI)**	***p***
PAR per 10-unit increase		1.14 (0.99–1.30)	0.060	1.22 (1.04–1.43)	0.014
**(B)**					
**Endpoint**	**Original HR**	**Median bootstrap HR**	**Bootstrap 95% CI**	**Proportion HR > 1 (%)**	
Overall Survival	1.95	1.75	0.55–9.52	82.2	
Progression-Free Survival	2.41	2.26	0.66–10.83	88.4	

Abbreviations: CI, confidence interval; HR, hazard ratio; PAR, platelet-to-albumin ratio. Continuous PAR values were incorporated into the adjusted multivariable Cox proportional hazards models as per a 10-unit increase variable. Footnote: Internal validation was performed using 1000 bootstrap resampling iterations applied to the final multivariable Cox proportional hazard models incorporating PAR status.

## Data Availability

The datasets generated and/or analyzed during the current study are not publicly available but may be obtained from the corresponding author upon reasonable request, subject to institutional review and approval by the Department of Clinical Oncology, University of Health Sciences Antalya Training and Research Hospital.

## Abbreviations

The following abbreviations are used in this manuscript:

CAR	C-reactive protein-to-albumin ratio
CI	Confidence interval
ECOG	Eastern Cooperative Oncology Group
IDH	Isocitrate dehydrogenase
IPCW	Inverse probability of censoring weighting
IQR	Interquartile range
LAR	Lactate dehydrogenase-to-albumin ratio
MGMT	O-6-methylguanine-DNA methyltransferase
MRI	Magnetic resonance imaging
NLR	Neutrophil-to-lymphocyte ratio
OS	Overall survival
PAR	Platelet-to-albumin ratio
PFS	Progression-free survival
PIV	Pan-immune inflammation value
RANO	Response Assessment in Neuro-Oncology
ROC	Receiver operating characteristic
SII	Systemic immune-inflammation index
SIRI	Systemic inflammation response index
VIF	Variance inflation factor
WHO	World Health Organization
